# Peptide ligand recognition by G protein-coupled receptors

**DOI:** 10.3389/fphar.2015.00048

**Published:** 2015-03-16

**Authors:** Brian E. Krumm, Reinhard Grisshammer

**Affiliations:** Membrane Protein Structure Function Unit, National Institute of Neurological Disorders and Stroke – National Institutes of HealthRockville, MD, USA

**Keywords:** neurotensin receptor, peptide agonist, peptide GPCRs, GPCR structure, chemokine receptors, opioid receptors, protease activated receptors

## Abstract

The past few years have seen spectacular progress in the structure determination of G protein-coupled receptors (GPCRs). We now have structural representatives from classes A, B, C, and F. Within the rhodopsin-like class A, most structures belong to the α group, whereas fewer GPCR structures are available from the β, γ, and δ groups, which include peptide GPCRs such as the receptors for neurotensin (β group), opioids, chemokines (γ group), and protease-activated receptors (δ group). Structural information on peptide GPCRs is restricted to complexes with non-peptidic drug-like antagonists with the exception of the chemokine receptor CXCR4 that has been crystallized in the presence of a cyclic peptide antagonist. Notably, the neurotensin receptor 1 is to date the only peptide GPCR whose structure has been solved in the presence of a peptide agonist. Although limited in number, the current peptide GPCR structures reveal great diversity in shape and electrostatic properties of the ligand binding pockets, features that play key roles in the discrimination of ligands. Here, we review these aspects of peptide GPCRs in view of possible models for peptide agonist binding.

## Introduction

G protein-coupled receptors are integral membrane proteins involved in many cellular processes including cell-to-cell communication, mediation of hormonal activity, and sensory transduction (Ji et al., 1998). Being of enormous clinical relevance, many GPCRs have been implicated as major therapeutic targets for the treatment of human diseases. Combining the recent explosion in GPCR structural biology with functional data highlights common principles for signal transduction, but more importantly also demonstrates many differences. Thus, despite our current knowledge, much still needs to be learned to fully comprehend the breadth and complexity of GPCR involvement in cell signaling.

G protein-coupled receptors recognize a large array of diverse natural ligands. If the ligand is an agonist, the GPCR catalyzes nucleotide exchange in cytoplasmic heterotrimeric GTP-binding proteins (G protein) leading to downstream events such as changes in the cAMP concentration in the cell. In addition, GPCRs have also been found to signal through arrestin-mediated cascades. These alternative G protein independent signaling pathways can be selectively stimulated alongside G protein activation, and a ligand’s efficacy can be “biased” more or less to different pathways (Violin and Lefkowitz, 2007).

Ligand/receptor/G protein interactions have been described by various models such as the classic ternary complex model (Kenakin, 2001). Central is the notion that GPCRs adopt different conformations, which do or do not allow productive interaction with the respective G protein. Simplified, the GPCR can be in a non-signaling, inactive state (R) by binding an inverse agonist, or it can be in a signaling-competent, active conformation (R*) with an agonist bound, catalyzing nucleotide exchange at the G protein. Today, GPCRs are no longer thought to be simple two-state switches (R or R*, although rhodopsin may come close to this definition) but are able to sample many conformations (Yao et al., 2009). Particular ligands can achieve varying efficacies by stabilizing a particular receptor conformation that can interact with G proteins and arrestins to varying degrees. Likewise, the presence of a particular intracellular signaling partner can also stabilize a given receptor conformation. Describing the structural basis for allosteric modulation and signaling bias (Katritch et al., 2013) is still one of the great challenges in GPCR structural biology.

For this review, we define inactive receptor states as GPCR conformations that are signaling incompetent, i.e., do not activate the G protein. Inactive receptor conformations may be stabilized by inverse agonists (Cherezov et al., 2007), but structures of agonist-occupied inactive GPCRs have also been reported (Rosenbaum et al., 2011; Egloff et al., 2014). Active-like conformations are activation intermediates, bound to agonist but not to G protein, with features characteristic for active GPCRs such as an outward-tilted transmembrane helix 6 at the cytoplasmic surface (White et al., 2012). An active receptor conformation is capable of catalyzing nucleotide exchange at the G protein, stabilized by both the agonist and G protein (Rasmussen et al., 2011b).

Our knowledge about GPCR structures has advanced tremendously over the past several years. We now have GPCR structures from classes A, B, C, and F. These structures are in complex with antagonists or inverse agonists, with agonists, and with G protein or G protein-mimicking antibodies. Thus they represent examples of inactive and active-like GPCR states and one distinct G protein signaling conformation of a receptor in complex with a heterotrimeric G protein (Rasmussen et al., 2011b).

Most of the known GPCR structures belong to the α group of the rhodopsin-like class A. The α group receptors characteristically bind small ligands within their transmembrane core. Fewer structures are available from the β, γ, and δ groups which include peptide binding GPCRs, the focus of this review. Current peptide GPCR structures include the NTSR1 (White et al., 2012; Egloff et al., 2014; β group); the DOR (Granier et al., 2012; Fenalti et al., 2014), KOR (Wu et al., 2012), MOR (Manglik et al., 2012), and the related NOP (Thompson et al., 2012; γ group); the chemokine receptors (CXCR4 Wu et al., 2010 and CCR5 Tan et al., 2013; γ group); and the PAR1 (Zhang et al., 2012; δ group). All peptide GPCRs, with the exception of NTSR1, have been crystallized in complex with non-peptidic drug-like antagonists in their inactive conformations. CXCR4 has also been crystallized in the presence of a cyclic peptide antagonist. NTSR1 has been co-crystallized with the peptide agonist NTS, both in an active-like conformation (White et al., 2012) and in an inactive state at the cytosolic domain (Egloff et al., 2014).

Peptide GPCRs bind agonists of a wide range of sizes, from a few amino acid residues in length to small proteins. In this review, we will discuss our knowledge of peptide GPCR structures with focus on their ligand binding pockets and ligands, and we will analyze those aspects in view of possible models for peptide agonist binding.

## Neurotensin Receptor 1

Neurotensin is a 13-amino-acid peptide (Carraway and Leeman, 1973) that is found in the nervous system and in peripheral tissues, where it functions as both a neurotransmitter and a hormone through activation of NTSR1. NTS shows a wide range of activities and has been implicated in Parkinson’s disease and the pathogenesis of schizophrenia, and the growth of cancer cells (Kitabgi, 2002). The crystal structure of NTSR1 has been determined in an active-like conformation (no G protein present) in complex with the peptide agonist NTS_8-13_, the C-terminal portion of NTS mediating agonist-induced activation of NTSR1 (White et al., 2012). This makes the NTSR1 structure distinct from the structures of other peptide GPCRs, which have all been crystallized in inactive states. NTSR1 has also been crystallized in the presence of agonist but in an inactive state at the cytosolic domain, lacking active-like characteristics (Egloff et al., 2014). Whilst several GPCRs have been crystallized with small molecule agonists (Choe et al., 2011; Rasmussen et al., 2011b; Warne et al., 2011; Xu et al., 2011), the NTSR1 structures represent to date the only example of a GPCR bound to a peptide agonist.

**FIGURE 1:**
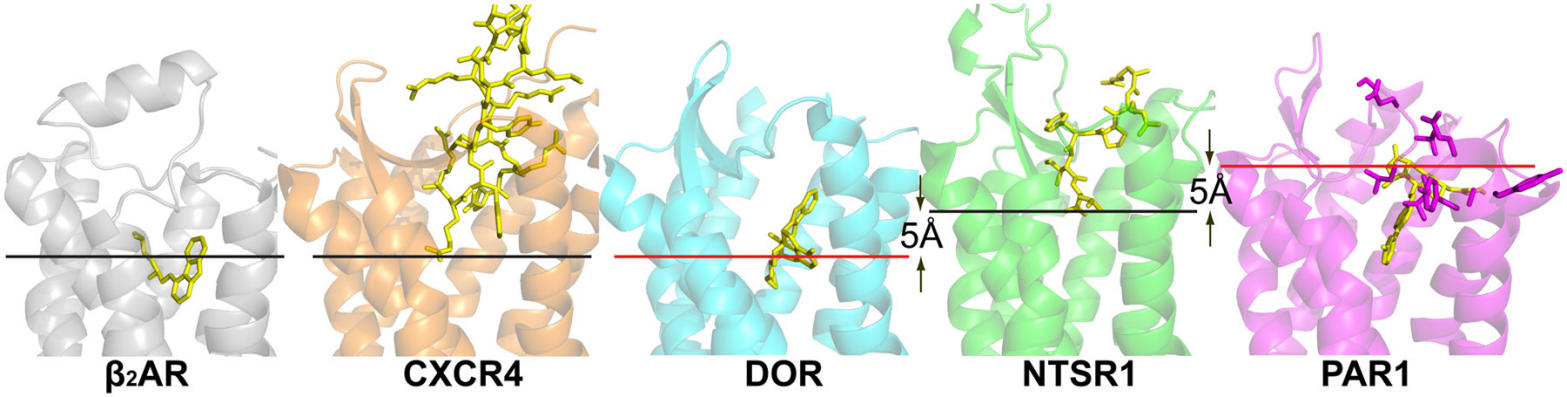
**Crystal structures of peptide receptors**. Receptors were aligned in PyMol. Ligands are shown as yellow sticks, receptors are shown as cartoons. CXCR4 with the cyclic peptide antagonist CVX15 (PDB code 3OE0), DOR with the morphinan antagonist naltrindole (PDB code 4EJ4), NTSR1 with the peptide agonist NTS_8-13_ (PDB code 4GRV), and PAR1 with the antagonist vorapaxar (PDB code 3VW7). For comparison, the α group member β_2_-adrenergic receptor with the partial inverse agonist carazolol (PDB code 2RH1) is shown. Red lines indicate the putative depth of peptide ligand binding as discussed in the review; black lines indicate the depth of ligand binding as seen in the respective structures. Residues of PAR1, implicated in tethered ligand binding, are shown as purple sticks.

The NTSR1 ligand pocket (White et al., 2012) is open and solvent exposed as that found in the opioid receptors and chemokine receptors, but unlike that of PAR1 (see below), with the NTSR1 N-terminus covering a small part of the ECL1 while interacting with ECL2. The peptide agonist binds to NTSR1 in an extended conformation, nearly perpendicular to the membrane plane, with the C-terminus oriented toward the receptor core (White et al., 2012) that is in the opposite orientation proposed for opioid peptides. There is a striking difference between the binding mode of NTS_8-13_ compared to the binding mode of small endogenous agonists. The NTS_8-13_ binding cavity is located near the receptor surface. Thus NTS_8-13_ does not penetrate the receptor deeply (**Figure 1**) placing the C-terminus of NTS_8-13_ over 5 Å away from small molecule agonist moieties seen in other GPCR structures. The mode of activation of NTSR1 may thus be subtly different from that of other GPCRs.

There is charge complementarity between NTS_8-13_ and its binding pocket with the positively charged arginine side chains of the ligand (Arg^8^–Arg^9^) facing the electronegative rim of the binding site, whereas the negatively charged carboxylate of Leu^13^ resides in an electropositive environment. There are also extensive van der Waals interactions between NTS_8-13_ and the receptor; key NTSR1 residues are in contact with NTS via hydrogen bonds and salt bridges. It is remarkable that only three out of eight hydrogen bonds are made between the side chains of NTS_8-13_ and the receptor, with the bulk of receptor-ligand contacts being van der Waals interactions.

## Opioid Receptors (DOR, KOR, MOR, NOP)

The classical opioid receptors DOR, KOR, and MOR, and the related NOP, play important roles in the central nervous system, regulating pain perception and mood (Pasternak, 2014). The structures of all four opioid GPCRs, in complex with subtype specific non-peptide antagonists, have been determined in their inactive conformations (Granier et al., 2012; Manglik et al., 2012; Thompson et al., 2012; Wu et al., 2012; Fenalti et al., 2014). The ligand binding pockets are wide open and solvent exposed, with the lower part being highly conserved among opioid receptors, and the upper part being diverse conferring subtype specificity. Thus the opioid receptor structures provided insight into the ‘message-address’ concept (Lipkowski et al., 1986) in which the ligand consists of two distinct parts with information about efficacy (message, in contact with the lower portion of the binding pocket) and selectivity (address, upper part of binding pocket). Many opioid antagonists (DOR specific naltrindole, KOR specific JDTic; MOR specific β-funaltrexamine) display common features such as a phenolic hydroxyl in close proximity to a positive charge (Granier et al., 2012; Manglik et al., 2012; Wu et al., 2012) resembling the N-terminal tyrosine residue of endogenous opioid peptides, for example endorphins, enkephalins, and dynorphins. The NOP specific compound C-24 has a benzofuran head group lacking the hydroxyl group (Thompson et al., 2012) reminiscent of the N-terminal phenylalanine of the nociceptin peptide. All the determined structures have an antagonist bound deep within the binding pocket at similar positions as agonists and antagonists in the β-adrenergic receptor, forming ionic interactions with an aspartate residue (Asp^3.32^) conserved in all opioid receptors, suggesting an essential role of Asp^3.32^ in anchoring positively charged ligands (Wu et al., 2012). Additional interactions between binding pocket residues and antagonists involve a water-mediated hydrogen bond network linking the antagonist phenolic hydroxyl to a conserved histidine residue (His^6.52^) in the classical opioid receptors (Granier et al., 2012; Manglik et al., 2012; Wu et al., 2012).

**FIGURE 2:**
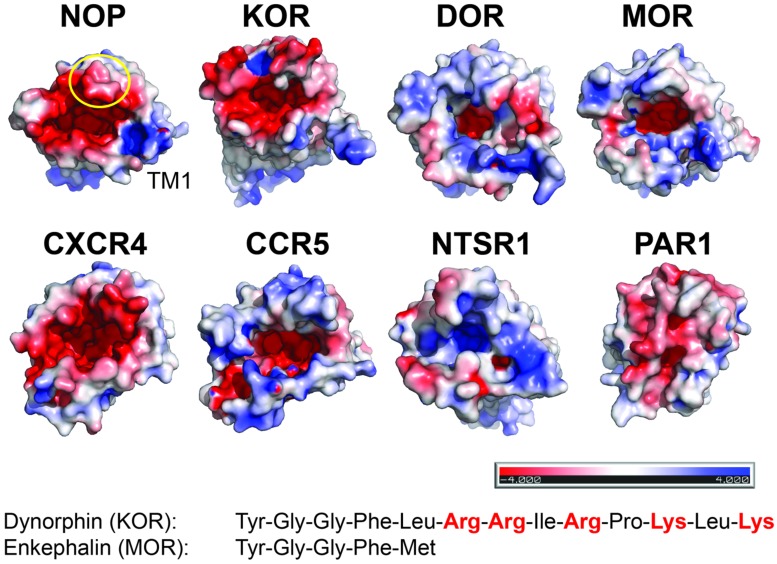
**Electrostatic surface properties contribute to discrimination between peptide ligands**. View from the extracellular side. The receptor surfaces are colored according to their electrostatic potential (scale bar -4 kTe^-1^ to +4 kTe^-1^; red, negative; blue, positive; PyMol using APBS tools). NOP (PDP code 4EA3); KOR (PDB code 4DJH); DOR (PDB code 4N6H); MOR (PDB code 4KDL); CXCR4 (PDB code 3OE0); CCR5 (PDB code 4MBS); NTSR1 (PDB code 4GRV); PAR1 (PDB code 3VW7). For orientation, the position of transmembrane helix 1 (TM1) and ECL2 (circle) are indicated in NOP. Examples of peptides for opioid receptors highlight the presence or absence of positive charges.

There are currently no structures of opioid receptors in complex with a peptide agonist or peptide antagonist. However, the binding mode and the similarity of features between non-peptidic antagonists and opioid peptides suggest that the N-termini of the opioid peptides might penetrate deeply into their respective receptors (**Figure 1**). Opioid peptides show great diversity in their chemical properties. For example, enkephalins are short peptides lacking charged amino acid side chains, whereas dynorphins and nociceptin are longer peptides with several Arg and Lys residues in their C-termini (**Figure 2** inset). Site-directed mutagenesis studies suggest that the MOR selective synthetic peptide agonist [D-Ala2,N-MePhe4,Gly-ol5] enkephalin makes both polar and non-polar contacts with the receptor (Seki et al., 1998; Manglik et al., 2012), reflecting the lack of highly charged surfaces in the MOR ligand binding site. In contrast, electrostatic surface potentials of NOP and KOR reveal striking differences compared to those of DOR and MOR (**Figure 2**). NOP and KOR have highly acidic patches at the extracellular side which likely form contact points for the basic C-termini of nociceptin and dynorphins. Molecular docking of the peptide antagonist UFP-101 into the NOP binding pocket corroborates that all six basic amino acids of the peptide are in contact with the acidic residues of ECL2 at the binding pocket entrance (Thompson et al., 2012).

## Chemokine Receptors (CXCR4, CCR5)

Chemokine receptors and their peptidic ligands, chemokines, are implicated in the migration of many cell types and constitute therapeutic targets owing to their role in many human disorders (Baggiolini, 1998). In addition, the chemokine receptors CXCR4 and CCR5 have been identified as HIV-1 co-receptors via the viral envelope glycoprotein gp120 (Berger et al., 1999). The structures of CXCR4 (Wu et al., 2010) and CCR5 (Tan et al., 2013) have been solved in complex with small drug-like inhibitors; CXCR4 has also been crystallized in complex with the 16 residue cyclic peptide inhibitor CVX15, an analog of the horseshoe crab peptide polyphemusin. Comparison of the CXCR4 and CCR5 structures provide clues about the determinants for chemokine binding and HIV-1 co-receptor selectivity.

CXC chemokine receptor 4 has been co-crystallized with the small-molecule antagonist IT1t, an isothiourea derivative, and the peptide CVX15. The ligand binding cavity is wide open although the entrance to the CXCR4 ligand binding pocket is partially covered by the receptor N-terminus and ECL2. The overall structures of CXCR4 with IT1t and with CVX15 are similar; however, the binding of the much larger CVX15 peptide caused some conformational differences compared to the CXCR4-IT1t structures. CXCR4 is activated by the chemokine CXCL12, and a two-site model of chemokine binding has been suggested separating the binding and signaling functions of chemokine ligands: the chemokine globular domain is thought to bind the receptor N-terminus and ECLs (site one) defining affinity and specificity, whereas the disordered N-terminal domain is thought to penetrate into the receptor helical core (site two) controlling receptor signaling. The IT1t and CVX15 complexes of CXCR4 may point to site two, with the CVX15 peptide residues Arg^1^ and Arg^2^ possibly indicating the depth of binding of the N-terminus of CXCL12 (**Figure 1**) whose residue Lys^1^ has been implicated in direct involvement in receptor activation.

CC chemokine receptor 5 has been co-crystallized with the inhibitor Maraviroc, an approved drug for the treatment of HIV-1 infection. The CCR5 binding pocket is more open than that of CXCR4. Maraviroc binding is distinct from the proposed major recognition sites for chemokines and the viral glycoprotein gp120, providing insight into allosteric inhibition of chemokine signaling and viral entry (Tan et al., 2013).

The third variable loop V3 of gp120 adopts a β-hairpin structure and has been shown to play a major role in cellular tropism and co-receptor specificity (Berger et al., 1999). Several acidic residues in the binding pocket of CXCR4 have been reported to be critical for HIV-1 infectivity. Interestingly, these acidic residues are substituted by uncharged residues in CCR5 resulting in different electrostatic surface potentials of the structures (**Figure 2**). This difference may correlate with the different charge properties of the V3 loops of X4- and R5-tropic viruses: X4-tropic viruses have a more positively charged V3 region complementary to the more negative surface of CXCR4, whereas V3 loops of R5-tropic viruses are less positively charged (Tan et al., 2013). Thus the structural features of CXCR4 and CCR5 highlight the possible importance of the net charge of the gp120 V3 loops for co-receptor selectivity.

## Protease Activated Receptor 1

Protease Activated Receptors are central to signaling through coagulation proteases. The proteases cleave the N-terminal receptor exodomain exposing a new tethered peptide agonist ligand irreversibly activating the respective PAR. A well-studied system is PAR1 activation by thrombin (Coughlin, 2000). The crystal structure of inactive PAR1 in complex with the antagonist vorapaxar has been solved (Zhang et al., 2012) providing insight how a small compound inhibits activation of PAR1 by the tethered ligand. Compared to opioid receptors, the vorapaxar binding pocket extends closer to the extracellular receptor side but is not well exposed to the aqueous solvent. This is in part due to the central location of ECL2 that covers the extracellular-facing portion of vorapaxar. Given the occluded access of the binding pocket from outside, reminiscent of the rhodopsin and the sphingosine-1-phosphate receptor (S1P_1_) structures, the lipophilic vorapaxar may enter PAR1 through the lipid bilayer (Zhang et al., 2012) in a similar way as proposed for retinal channeling to opsin (Park et al., 2008) or for lipid to the S1P_1_ receptor (Hanson et al., 2012).

Thrombin cleaves PAR1 to generate a new N-terminus starting at Ser^42^, which can bind, and activate PAR1. The PAR1-vorapaxar structure does not provide insight how the agonist peptide gains access to its binding site. However, the structure is consistent with mutagenesis data proposing that the agonist peptide is in contact with superficial receptor areas rather than reaching deep into the transmembrane core (Gerszten et al., 1994; Zhang et al., 2012). Specifically ECL2 has been implicated in the binding of the tethered agonist (Nanevicz et al., 1996; **Figure 1**). The surface location of these residues may thus imply that superficial interaction between the tethered PAR1 N-terminus and extracellular receptor loops suffices to activate PAR1. Alternatively, the initial binding of the tethered peptide agonist may lead to deeper penetration of the receptor N-terminus into the transmembrane core through a series of conformational changes of PAR1 (Zhang et al., 2012).

## Possible Binding Modes of Peptides

Peptide receptors bind peptide ligands of a wide range of sizes, from a few amino acids in length such as enkephalin (5 residues), to longer peptides such as NTS (13 residues) and nociceptin (17 residues), to small proteins such as chemokines (∼90 residues). The distinct features of peptide ligands thus necessitate complementary receptor characteristics to promote a specific signaling event.

Although most peptide receptor structures are not with peptide ligands, they nevertheless provide information on putative binding modes of peptides. First, peptides may reach deeply into the receptor core (opioid peptides); bind closer to the receptor surface NTS; or are in contact with superficial receptor areas (tethered PAR1 ligand). Chemokine ligands may combine all of those aspects. As minute changes in the buried binding sites for small drug-like agonists trigger the larger conformational changes on the intracellular receptor surface upon activation (Rasmussen et al., 2011a), additional structures of peptide GPCRs are eagerly awaited to rationalize how binding of a peptide agonist closer to or on the receptor surface causes the intracellular helical rearrangements of the activated state thought to be conserved in all class A GPCRs.

Second, matching electrostatic properties between peptide ligand and binding pocket (or their absence) allows discrimination between ligands. For example, KOR has highly acidic patches at the extracellular side, which likely interact with the basic C-terminus of dynorphin; in contrast, MOR lacks such a pronounced negative surface potential reflecting the uncharged nature of enkephalins.

Third, subtype specificity and ligand affinity are given by the complementary shape and property of the binding site. Because of the larger size of peptides compared to small-molecule compounds, extensive van der Waals contacts would provide additional discriminatory aspects for peptide binding characteristics. For example, the hexapeptide NTS_8-13_ has 34 potential intermolecular interactions with eight hydrogen bond-mediated contacts. It is striking that only three out of the eight hydrogen bonds are made between the side chains of NTS_8-13_ and the receptor, with the bulk of receptor-ligand contacts being van der Waals interactions (White et al., 2012).

Fourth, most peptide receptor structures show inactive, signaling incompetent conformations. Only the structure of NTSR1 represents an active-like, agonist-bound state (White et al., 2012). As further structural changes are expected to occur upon engagement of an agonist-occupied receptor with G protein (Rasmussen et al., 2011b), additional structures of GPCRs bound to peptide agonists and G protein are needed to define common principles and also differences in the signaling conformations of peptide receptors compared to receptors from the α group of class A that bind small agonists within the transmembrane core.

## Authors’ Note

While the manuscript was in submission, the crystal structures of the orexin 2 receptor in complex with the non-peptide inhibitor suvorexant (Yin et al., 2014), and of the chemokine receptor CXCR4 in complex with a viral chemokine (Qin et al., 2015) have been published. The coordinates for a δ-opioid receptor bound to a bifunctional peptide (Fenalti et al., 2014) have been released in the Protein Data Bank.

## Abbreviations

CCR5: CC chemokine receptor 5
CXCR4: CXC chemokine receptor 4
DOR: δ-opioid receptor
ECL: extracellular loop
GPCR: G protein-coupled receptor
KOR: κ-opioid receptor
MOR: μ-opioid receptor
NOP: nociceptin/orphanin FQ receptor
NTS: neurotensin
NTSR1: neurotensin receptor 1
PAR1: protease-activated receptor 1
